# Spatiotemporal Population Genomics of the Invasive Whitefly *Bemisia tabaci* MED in China: Implications for Surveillance and Sustainable Control

**DOI:** 10.3390/insects16090975

**Published:** 2025-09-17

**Authors:** Kun Yang, Hongran Li, Dong Guo, Zuowen Sun, Fujun Li, Dong Chu

**Affiliations:** 1 Shandong Engineering Research Center for Environment-Friendly Agricultural Pest Management, College of Plant Health and Medicine, Qingdao Agricultural University, Qingdao 266100, China; yangkun@qau.edu.cn; 2 Shandong Province Centre for Bioinvasions and Eco-Security, Qingdao 266100, China; 3 Key Laboratory of Integrated Pest Management on Crops in East China, Ministry of Agriculture and Rural Affairs, Nanjing Agricultural University, No. 1 Weigang, Nanjing 210095, China; 4 Guangdong Laboratory of Lingnan Modern Agriculture, Shenzhen, Genome Analysis Laboratory of the Ministry of Agriculture, Agricultural Genomics Institute at Shenzhen, Chinese Academy of Agricultural Sciences, Shenzhen 518000, China; lihongran@caas.cn; 5 General Station for Plant Protection of Shandong Province, Jinan 250014, China; guod319@163.com (D.G.); sunzuowen@163.com (Z.S.); 6 Shandong Weifang Academy of Agricultural Sciences, Weifang 261000, China; fujunli76@163.com

**Keywords:** *Bemisia tabaci* MED, population genetics, biological invasion, invasive species, dispersal, gene flow

## Abstract

*Bemisia tabaci* MED is one of the most invasive and destructive agricultural pests worldwide, posing a serious threat to crop production and biosecurity. Understanding its spatiotemporal population dynamics and genetic structure is critical for early detection, effective surveillance, and sustainable management. Previous studies have shown that *B. tabaci* MED in China has a high genetic structure and an unstable genetic composition. The annual genetic dynamics of the *B. tabaci* MED population have not been investigated throughout the outbreak phase that began in 2008. Here, we report the use of 2b-RAD sequencing to estimate the spatial and temporal genetic structure of *B. tabaci* MED in Shandong Province over several years. We examined 198 individuals from five sites over four years (2008, 2013, 2015, and 2017). Although populations showed generally low within-population diversity (Shannon I ≤ 0.407) and a high gene flow, clear temporal differentiation emerged between the early invasion phase (2008) and later outbreak years (2013–2017). Furthermore, specific populations, notably 2017 Liaocheng and Zaozhuang, retained distinct genetic signatures compared with other regions, suggesting localized founder effects or adaptation. Our study underscores the importance of integrating high-resolution genomic tools into invasive pest surveillance programs, and understanding this heterogeneity is critical for targeted surveillance, quarantine prioritization, and sustainable management strategies.

## Simple Summary

*Bemisia tabaci* MED (Mediterranean), a globally invasive agricultural pest, has severely impacted Chinese agriculture since its 2003 introduction. This study employs 2b-RAD sequencing to analyze the genetic structure of whitefly populations across Shandong Province during 2008–2017. We reveal spatiotemporal heterogeneity across populations, which could be affected by ecological factors. Temporal analysis shows genetic shifts between initial- (2008) and outbreak-phase populations, especially for whiteflies in the Zaozhuang distribution. STRUCTURE and DAPC analysis showed a spatial heterogeneity of different geographical populations. Understanding this heterogeneity is critical for targeted surveillance, quarantine prioritization, and sustainable management strategies.

## 1. Introduction

The genetic structure of a pest population reflects the interaction between genetic drift, mutation, migration, and selection [1,2,3]. Understanding the genetic structure of invasive alien populations can provide insights into the processes that shape their population dynamics and responses to novel abiotic and biotic conditions [4,5]. An understanding of population structure can also provide insights into appropriate pest management strategies [6]. Invasive species often evolve rapidly, but this depends on additive genetic variation [7]. Thus, gaining a better understanding of the spatial and temporal genetic structure of the whitefly *Bemisia tabaci* MED (Hemiptera: Aleyrodidae), which invaded China in 2003 [8], should elucidate aspects of the ecology of this pest and assist in the development of strategies to manage it.

Population studies of genetic variation in space and time can provide insights into the processes that shape successful biological invasion [5]. A high level of population structuring may indicate the adaptation of populations to their local environments [9] or random founder effects as a species expands [9]. In contrast, weak structuring and high genetic diversity may indicate high tolerance to environmental change and a large potential to adapt to novel environmental pressures [10]. Many invasive pests arise from multiple introductions, which can enhance the likelihood of their establishment and help them adapt and spread [11,12]. Introduced populations can benefit from the admixture of genes from many source populations, which, in turn, increases additive genetic variation, creates novel genotypes, and masks deleterious mutations [10].

In agroecosystems, it is important to determine the genetic structure of pest populations to design and optimize sustainable pest management programs [4,13]. For instance, population-level gene flow plays a critical role in the evolution of insect resistance to pesticides and transgenic crops [14]. The *B. tabaci* species complex, including *B. tabaci* MED, has evolved resistance to many insecticides [15,16]. Therefore, understanding the population structure of China is important for pest management.

*Bemisia tabaci* is a species complex consisting of at least 36 morphologically indistinguishable but reproductively isolated cryptic species, including Mediterranean (MED) species [17]. They are polyphagous invasive pests that damage many crops. *Bemisia tabaci* MED (formerly known as biotype Q) invaded many provinces over 10 years, following its first detection in China in 2003 [7], and replaced many indigenous species, especially in North China [18,19,20]. The invasion history of *B. tabaci* MED in China was considered to have three stages: early colonization (2003), spread (2004–2007), and outbreak (after 2008) [12]. The genetic structure of *B. tabaci* MED after its initial introduction and subsequent spread in China was examined and a high genetic structure was detected among the populations sampled throughout China [21].

Understanding how genetic variation is distributed at different spatial and temporal scales is critical because genetic variation influences the outcome of species invasions [8]. Genetic turnover has often been observed in *B. tabaci* [22,23], which may be fairly common given the pressures of changing habitats and environments, as well as the influence of changing patterns of insecticide applications [24].

Gene flow resulting from the dispersal of pests influences population structure. Species in the *B. tabaci* complex do not always disperse passively [25]. They have been shown to have both migratory and trivial flying morphs [26]. The *B. tabaci* species complex is also spread on planting materials, cut flowers, fruit, and vegetables [27]. Thus, human activities also influence gene flow at local and geographic scales. It is unclear how and over what spatial scale *B. tabaci* MED might respond to yearly changes in the landscapes they inhabit.

In a previous study, we identified the successful invasion and rapid adaptation of *B. tabaci* MED globally by rapidly expanding gene families and positively selected genes [28]; however, the population structure of *B. tabaci* MED in China, especially in Shandong Province, has not been fully explored. Here, we report the changing population structure of *B. tabaci* MED in Shandong Province, China, during the outbreak phase, using 2b-RAD sequencing. The 2b-RAD sequencing method is notable for its low cost, simple protocol, uniform tags, adjustable marker numbers, and compatibility with non-model samples, making it ideal for use in insect population genomics [29,30,31,32].

Previous studies on the population genetics of *B. tabaci* MED in China have primarily relied on mitochondrial DNA or microsatellite markers, which offer limited resolution for fine-scale temporal analysis [19,20,21,22,23]. Furthermore, while these studies captured the initial invasion and spread, the annual genetic dynamics during the critical outbreak phase (post-2008) remain unexplored at a genomic scale. This study addresses this gap by employing high-resolution 2b-RAD sequencing to genotype populations from five regions in Shandong Province across four key years (2008, 2013, 2015, and 2017). This approach allows us to quantify spatiotemporal genetic changes with unprecedented detail, providing insights into the decadal-scale evolutionary dynamics of an invasive pest post-establishment, which is critical for refining surveillance and management strategies.

## 2. Materials and Methods

### 2.1. Sample Collection of Bemisia tabaci MED in Shandong, China

**Figure 1:**
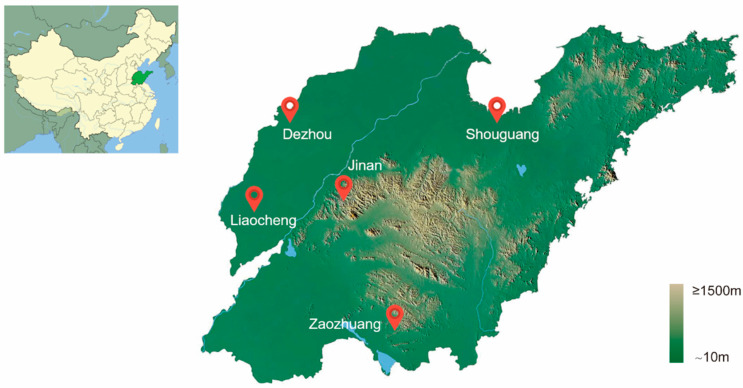
Sample sites for collecting *Bemisia tabaci* MED in Shandong province, China.

Sampling was conducted across Shandong Province, China (Figure 1), a region characterized by a continental monsoon climate with warm, humid summers and cold, dry winters. This area is a major center for vegetable and crop production. *Bemisia tabaci* adults were collected from greenhouses and open fields in five municipal regions—Dezhou, Liaocheng, Jinan, Shouguang, and Zaozhuang—in the years 2008, 2013, 2015, and 2017 (Table 1). Collections were made from various host plants, including cotton, eggplant, and other vegetables (Supplementary Table S1) using a random sampling strategy with an aspirator. The specimens were preserved in 99.8% ethanol before DNA extraction. A total of 198 individuals representing all sampling years and locations were selected for mitochondrial gene sequencing to confirm the species’ identity.

### 2.2. 2b-RAD Sequencing and Genotyping of Bemisia Tabaci MED

Total DNA was extracted from individual whiteflies using the TIAMamp Micro DNA Kit (Tiangen, Beijing, China) according to the manufacturer’s protocol. 2b-RAD sequencing and genotyping were performed by Qingdao OE Biotech, Ltd. (Qingdao, China). The libraries were constructed using the 2b-RAD protocol [32]. Briefly, library preparation began with the digestion of DNA samples. The BsaXI restriction enzyme (New England BioLabs, Ipswich, MA, USA) was used to prepare the RAD libraries. Next, library-specific adaptors and digestion products were linked using T4 DNA ligase (New England Biolabs). The ligation products were amplified by PCR, and the target bands were excised from a 2% agarose gel. Finally, the paired-end RAD tags were sequenced on an Illumina HiSeq Xten platform (Illumina, San Diego, CA, USA). Raw sequence reads were trimmed to remove adaptors, and the terminal 2 bp positions were discarded to eliminate artifacts that might have arisen from ligation. Ambiguous bases (N) or reads of low quality (>10 bp with quality less than Q20) were removed. SNPs were determined and genotypes were called using a maximum-likelihood statistical model implemented in the Stacks v1.32 software [33].

### 2.3. SNP Filtering and Genetic Diversity Analyses

The genotype data contained information for each locus and individual. The number of primary SNP loci was 23,634, and all 198 individuals could be genotyped. Plink version 1.07 [34] was used to filter SNPs for genetic analysis. SNPs were filtered to meet the following criteria: (a) SNPs included in at least 80% of the sample population, (b) SNPs with a minor allele frequency (MAF) higher than 0.05, and (c) loci with strong deviations from the Hardy–Weinberg equilibrium (HWE, *P* < 0.0001) were removed. The final filtered SNP dataset contained 1871 SNP loci and was used for all downstream analyses. The parameters for population genetic analyses, that is, the percentage of polymorphic loci (%poly), Shannon’s information index (I), observed heterozygosity (HO), expected heterozygosity (HE), and inbreeding coefficient (FIS), were estimated by using GenALEx v.6.5 [35,36]. The Hardy–Weinberg equilibrium (HWE) and excess and deficit heterozygosity were tested using GENEPOP (v. 4.2.1) [37]. Pairwise population fixation index (FST) values and associated P-values were also estimated via permutation in GENEPOP v.4.2.1.

### 2.4. Analysis of Molecular Variance and Mantel Test

Analysis of molecular variance (AMOVA) was used to characterize the genetic variation patterns and estimate the variance components. We also tested the effect of location (sampling site) within the sampling year as well as the effect of sampling year within the location. Using a hierarchical AMOVA, we were able to independently estimate the effects of year and geographic location. This analysis was performed using GenAlEx v.6.5. The significance was estimated using 999 permutations.

### 2.5. Analyses of Population Structure

The Bayesian approach was used to determine genetically distinct groups (or clusters) using the program STRUCTURE v.2.3.1 [38]. The length of the burn-in period was set to 10,000, and the number of MCMC replications after burn-in was 10,000. We set the K value from 1 to 10, and the number of iterations was 10 for each K. To estimate the group number, we used the online calculation [39]. We examined the change in Ln P(D) using the delta-K approach [40]. Because the STRUCTURE software showed the results of each of the 10 replications in the case of K = n, we used CLUMPP to average these results [41]. All data were visualized using DISTRUCT v.1.1 [42].

We also conducted a discriminant analysis of principal components (DAPC) in R v.3.6.3 [43] using the ‘adegenet’ package [44]. The DAPC can provide an efficient description of genetic clusters and facilitate the detection of complex population structures [45].

## 3. Results

### 3.1. Genetic Variation Within and Among Bemisia tabaci MED Populations in Shandong Province of China

Genetic variation within *B. tabaci* MED populations exhibited several consistent patterns: high polymorphism across loci coupled with low heterozygosity and a universal heterozygosity deficit, as detailed in Table 1. The percentage of polymorphic loci (%Poly) ranged from 81.67–87.60% among the 20 populations, indicating that most loci were polymorphic in all populations. Despite overall low heterozygosity, samples collected in 2008 (e.g., ZZ08, LC08) showed significantly lower genetic diversity than later years, reflecting residual founder effects, which describes the long-lasting genetic signature of a population bottleneck that occurs when a new population is established by a small number of individuals from a larger source population. Despite this, estimates of genetic diversity were relatively low, with Shannon’s information index (I) ranging from 0.339 in SG13 to 0.407 in SG17. The observed heterozygosity (HO) ranged from 0.163 (SG17) to 0.194 (SG08, LC08). The expected heterozygosity (HE) ranged from 0.198 (LC08) to 0.233 (SG17), which was greater than the HO value in every sample. Moderate levels of inbreeding (FIS > 0.05) were detected in 17 out of 20 populations, and there were significant departures from the Hardy–Weinberg equilibrium expectations (*P* < 0.05) in all populations, consistent with inbreeding or non-random mating.

The fixation index (FST) reflects the degree of genetic differentiation among populations. The global FST for the dataset was 0.0042. The estimated pairwise FST values ranged from −0.00446 to 0.0124 among the populations (Table 2 and Table S2). No significant genetic differentiation was detected among the populations collected from different locations in the same year (*P* > 0.05). However, when comparing populations across years, particularly between 2008 and the later years (2013, 2015, and 2017), significant genetic differentiation was observed (mean FST = 0.0032, *P* < 0.001). Abbreviations: LC, Liaocheng; ZZ, Zaozhuang; SG, Shouguang; DZ, Dezhou; JN, Jinan.

### 3.2. Population Structure of Bemisia tabaci MED

The AMOVA results showed that only 0.42% of the total genetic variation was accounted for by variation among the populations, and 20.33% of the genetic variation was attributable to differences within populations. When grouping populations by sample site, hierarchical AMOVA showed no significant effect of the sample site, but there was a significant effect of year nested within the sample site (Table 3). When grouping populations by year, a slight but marginally significant year effect was also observed (0.16% variance, *P* = 0.051) (Table 3).

**Figure 2:**
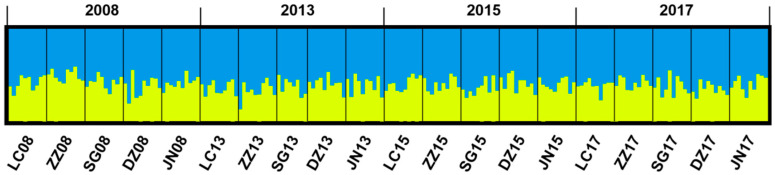
Bayesian analysis to determine suitable cluster (K) and estimated cluster proportion using STRUCTURE for *Bemisia tabaci* MED. For the assignment proportion in all populations, each individual is represented by a thin vertical line, which was partitioned into K segments that represented its estimated population group membership fractions.

The Bayesian clustering approach in STRUCTURE suggested that the highest likelihood value of K was obtained at K = 2 for 20 populations. The mean log-likelihood value decreased at K = 2 (Supplementary Figure S1). The pattern of admixture structure was similar among all populations (Figure 2).

**Figure 3:**
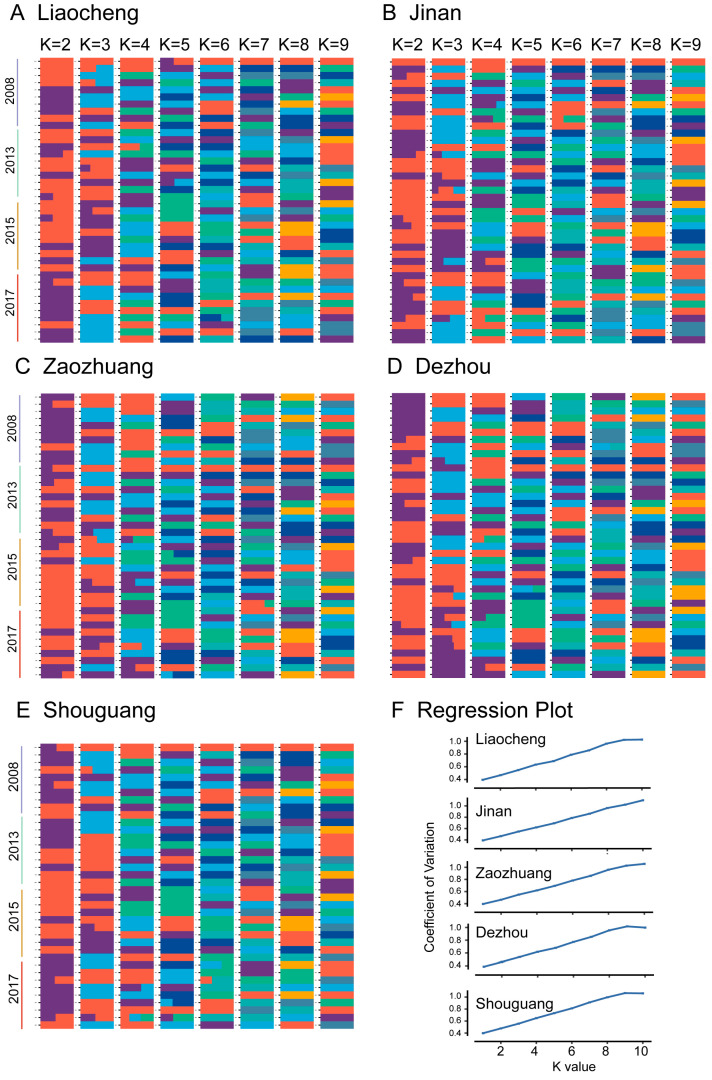
Genetic population structure of *Bemisia tabaci* MED populations in Shandong province, China. Map of structure of *Bemisia tabaci* MED populations in 5 municipal regions (K = 2 to 9), including Liaocheng (**A**), Jinan (**B**), Zaozhuang (**C**), Dezhou (**D**), and Shouguang (**E**), were shown, respectively, with different colors representing the components from the respective major groups in each sample population. Each area includes populations collected in the years 2008, 2013, 2015, and 2017. (**F**): the linear relationship between K value and CV in 5 locations populations.

When comparing the admixture structure of populations collected in the same city in different years, samples collected in 2013 and 2015 were similar in all populations within a single distribution; samples collected in 2008 in Liaocheng, Zaozhuang, and Dezhou, and samples collected in 2017 in Liaocheng were distinct from other populations (Figure 3), suggesting spatial and regional heterogeneity. When K-values increased, the coefficient of variation (CV) also increased in all populations within one distribution (Figure 3F).

**Figure 4:**
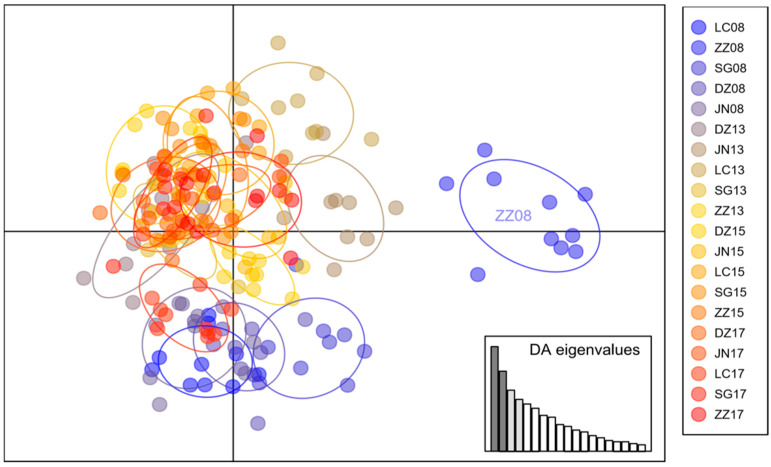
Results of discriminant analysis of principal components (DAPC) showing the clusters of 20 *Bemisia tabaci* MED populations in Shandong province, China.

Discriminant analysis of principal components (DAPC) showed a broad overlap among most populations (Figure 4), but population ZZ08 (Zaozhuang 2008), LC08 (Liaocheng 2008), and SG08 (Shouguang 2008) appeared distinct from the others.

**Figure 5:**
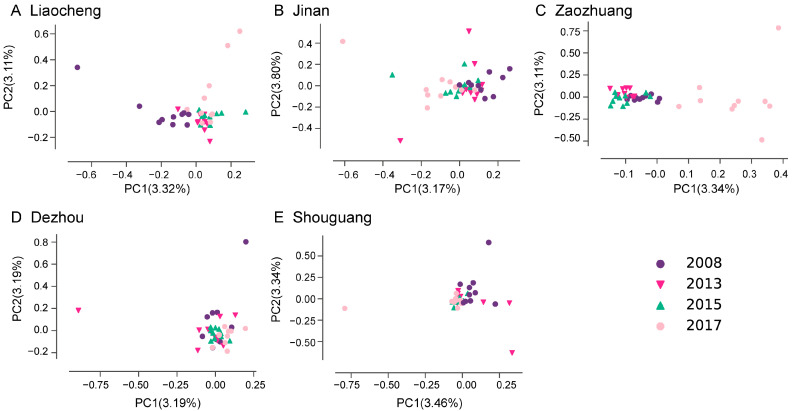
Discriminant analysis of principal components (DAPC) of *Bemisia tabaci* MED populations within individual municipal regions of Shandong Province, China. Results of discriminant analysis of principal components (DAPC) showing the clusters of populations within the same municipal region; in total, 5 areas including Liaocheng (**A**), Jinan (**B**), Zaozhuang (**C**), Dezhou (**D**), and Shouguang (**E**) were shown, respectively.

Populations collected in 2008 formed separate clusters, particularly in Liaocheng, Jinan, and Shouguang (Figure 5A,B,E). In Liaocheng and Zaozhuang, the populations sampled in 2017 were also distinct from those sampled in other years (Figure 5A,C).

## 4. Discussion

### 4.1. Temporal Genetic Heterogeneity Reveals Bemisia tabaci Med Invasion History

The genetic distinctiveness of 2008 populations likely reflect the immediate post-introduction stage, where multiple introduction sources or founder effects maintained residual structure [22], while the observed low genetic diversity (I ≤ 0.407) and heterozygosity deficits across *B. tabaci* MED populations aligned with patterns commonly reported in invasive species undergoing founder effects and rapid range expansion [9,22]. Weak temporal signals persisted, especially in Liaocheng and Zaozhuang, indicating localized founder events or micro-adaptation to agricultural niches. Notably, the high frequency of polymorphic loci contrasts with low heterozygosity, suggesting that allelic diversity may have been preserved through multiple introductions or admixture events [10], while subsequent bottlenecks or inbreeding eroded heterozygosity. This paradox warrants further investigation; while SNP markers capture genome-wide neutral variation, they may not reflect adaptive loci under selection in heterogeneous environments [46].

### 4.2. Spatial Genetic Heterogeneity Driven by Anthropogenic and Ecological Actors

While global FST was low (0.0042), STRUCTURE and DAPC consistently detected regional deviations in 2017 Liaocheng and Zaozhuang. These populations differed significantly from others, which may have resulted from restricted greenhouse populations overwintering, specific insecticide regimes, or other ecological effects causing localized selection [47,48,49,50]. The results were similar to our previous study based on the analysis of microsatellites of whiteflies [23]. In fact, microsatellites, with higher mutation rates, better resolve recent divergence, whereas SNPs reflect deeper demographic processes [51]. Notably, greenhouses likely serve as genetic reservoirs of *B. tabaci* MED during winter, enabling yearly recolonization of open fields [47,50,51]. This suggests that even in a highly connected landscape, small-scale heterogeneity persists and could seed future adaptive divergence.

### 4.3. Implications of Spatiotemporal Heterogeneity for Pest Management

The spatiotemporal genetic heterogeneity of *B. tabaci* MED underscores the need for dual-focused management strategies. A high gene flow, facilitating the spread of advantageous traits (e.g., insecticide resistance), necessitates region-wide coordination of resistance management programs. Conversely, localized genetic distinctiveness (e.g., in Liaocheng and Zaozhuang) requires targeted interventions, including prioritized genomic surveillance and regionally tailored chemical and biological control adapted to resistant variants and symbiont–host interactions. Management must therefore integrate broad resistance mitigation with localized responses to unique adaptive pressures.

Genetic backgrounds are important for development, resistance, as well as many other vital aspects of host insects [49,52,53,54,55,56]. For example, the symbiont *Cardinium* infection and titer in *B. tabaci* are largely influenced by genotypes of host whiteflies [53,54], while *Cardinium* infection is vital for the thermotolerance of *B. tabaci* [55]. Furthermore, the critical influence of genetic backgrounds is a widespread phenomenon; for instance, mutations in *Drosophila melanogaster* are known to drive neural circuit hyperconnectivity [56]. In total, integrating genomic scans for selection could identify loci under local adaptation, enabling predictive models of pest response to climate change or crop shifts. Finally, the role of non-crop hosts and greenhouse microhabitats as refugia requires attention, as these may sustain the genetic diversity critical for future invasions.

### 4.4. Limitations and Future Directions

Our strategic sampling targeted key invasion phases (initial spread vs. established outbreak) rather than consecutive years, creating a gap in 2010–2012. While this may have missed short-term fluctuations, the high gene flow and significant, sustained genetic differentiation observed between 2008 and all later outbreak years indicate a fundamental and lasting population shift. We are therefore confident that the chosen time points robustly capture the major decadal-scale genetic dynamics, and our conclusions remain valid. Future work with continuous annual sampling would be valuable to resolve finer-scale evolutionary processes.

Although this study focused on the host’s nuclear genome, the role of microbial symbionts must be considered for a holistic interpretation of *B. tabaci* MED’s invasion ecology. These endosymbionts can profoundly influence host biology, affecting insecticide resistance, thermotolerance, virus transmission, and fitness. Crucially, their infection frequencies and densities are often influenced by the host’s genetic background [52,53,54]. Therefore, the spatiotemporal genetic heterogeneity we observed could underpin parallel heterogeneity in the whitefly’s microbiome. For instance, a genetically distinct population (like ZZ17 or LC17) might harbor a different symbiotic consortium, which in turn could drive local adaptation—such as enhanced thermal tolerance conferred by *Cardinium* [55]—independent of the host nuclear genome. This potential interaction between host genotype and microbiome creates a multi-layered adaptive architecture. Future studies that integrate host genomics with microbiomic profiles are essential to unravel these complex interactions and their collective impact on the invasion success and management of this global pest.

Future work should integrate environmental data with genome–environment association analyses to further explore the occurrence of spatiotemporal heterogeneity of *B. tabaci* MED. Additionally, the 10-year sampling window may miss finer temporal dynamics; monthly sampling across seasons could reveal how agricultural practices modulate gene flow. Lastly, expanding the geographic scope to compare Shandong with other Chinese regions would clarify whether genetic spatiotemporal heterogeneity is a provincial or nationwide phenomenon.

## 5. Conclusions

Our study reveals that *Bemisia tabaci* MED populations in Shandong are not entirely homogeneous; rather, they exhibit temporal transitions and localized heterogeneity superimposed on high regional gene flow. Early invasion populations (2008) retained distinct genetic signatures, while later years reflected a more panmictic structure punctuated by localized differentiation in Liaocheng and Zaozhuang. These spatiotemporal heterogeneities elucidate the invasion complexity of *B. tabaci* MED, guiding precision surveillance and sustainable management.

## Figures and Tables

**Table 1:** Genetic diversity indices for *Bemisia tabaci* MED populations sampled from five locations in Shandong Province across one decade.

Sampling Year	Sampling Location	Population ID	%Poly	I	Ho	He	FIS
2008	Liaocheng	LC08	84.45	0.341	0.194	0.198	0.006
	Zaozhuang	ZZ08	85.84	0.352	0.193	0.207	0.036
	Shouguang	SG08	86.96	0.376	0.194	0.217	0.077
	Dezhou	DZ08	86.37	0.396	0.175	0.226	0.179
	Jinan	JN08	86.85	0.376	0.192	0.217	0.085
2013	Liaocheng	LC13	85.03	0.366	0.176	0.211	0.117
	Zaozhuang	ZZ13	85.52	0.377	0.167	0.214	0.172
	Shouguang	SG13	81.67	0.339	0.175	0.199	0.073
	Dezhou	DZ13	85.94	0.350	0.185	0.203	0.060
	Jinan	JN13	85.52	0.376	0.172	0.216	0.159
2015	Liaocheng	LC15	86.85	0.380	0.182	0.218	0.126
	Zaozhuang	ZZ15	87.33	0.369	0.187	0.213	0.092
	Shouguang	SG15	87.60	0.389	0.174	0.223	0.176
	Dezhou	DZ15	87.28	0.356	0.190	0.206	0.050
	Jinan	JN15	86.91	0.380	0.178	0.217	0.138
2017	Liaocheng	LC17	86.75	0.383	0.180	0.220	0.147
	Zaozhuang	ZZ17	86.53	0.368	0.185	0.212	0.097
	Shouguang	SG17	87.33	0.407	0.163	0.233	0.248
	Dezhou	DZ17	87.44	0.389	0.176	0.221	0.166
	Jinan	JN17	87.07	0.364	0.191	0.211	0.061

Notes: percentage of polymorphic loci (%poly), Shannon’s information index (I), observed heterozygosity (HO), expected heterozygosity (He), and inbreeding coefficient (FIS). There were 10 samples for each location, except for SG13, which had eight samples.

**Table 2:** Analysis of molecular variance (AMOVA) and average pairwise population differentiation (FST) among *Bemisia tabaci* MED populations grouped by sampling year.

Group Comparison	Average Fst	*P*-Value
Among all populations	0.0042	0.006
Among years (Global)	0.0032	<0.001
2008 vs. 2013	0.0031	<0.001
2008 vs. 2015	0.0052	<0.001
2008 vs. 2017	0.0041	<0.001
2013 vs. 2015	0.0011	0.112
2013 vs. 2017	0.0010	0.145
2015 vs. 2017	0.0015	0.087
Within same years (Spatial)	−0.0001–0.0020	>0.05

**Table 3:** Analysis of molecular variance (AMOVA) and Monte Carlo permutation test results of *Bemisia tabaci* MED population in Shandong Province.

Group of Populations	Source of Variation	df (Degree of Freedom)	Sum of Squares	Variance Components	Percentage Variation (%)	*P*-Value
Global	Among populations	19	5219.287	0.899	0.42	0.006
	Within populations	178	45,729.188	43.553	20.33	0.001
	Within individuals	198	33,620.500	169.801	79.25	0.001
Group by geographic	Among geographic groups	4	1111.149	0.049	0.02	0.361
	Among yearly populations within geographic groups	15	4108.138	0.858	0.40	0.021
	Within populations	178	45,729.188	43.553	20.33	0.011
	Within individuals	198	33,620.500	169.801	79.25	0.001
Group by years	Among yearly groups	3	909.059	0.339	0.16	0.051
	Among geographic populations within yearly groups	16	4311.158	0.634	0.30	0.044
	Within populations	178	45,729.188	43.553	20.32	0.006
	Within individuals	198	33,620.500	169.801	79.23	0.001

## Data Availability

Sequence data that support the findings of this study have been deposited in the NCBI with the primary accession code SUB15351231 (data of years 2008 and 2013) and SUB15358493 (data of years 2015 and 2017).

## Supplementary Materials

The following supporting information can be downloaded at: https://www.mdpi.com/article/10.3390/insects16090975/s1, Table S1. Summary of sample sources; Table S2. Pairwise FST values for all pairs of populations, all the values were insignificant (*P* > 0.05); Figure S1. (a) Delta k of Evanno et al. (2005) [40] across 10 replicates of STRUCTURE, where k = 2 is shown as the best fit of the data for the highest level of hierarchical genetic structure. (b) The mean lnP(D|K) and SD of 10 replicates of STRUCTURE runs for each k where the model of k = 2 is indicated as the best fit.
