# Supplementary material for: Spatiotemporal Population Genomics of the Invasive Whitefly Bemisia tabaci MED in China: Implications for Surveillance and Sustainable Control
Source: Insects. 2025 Sep 17;16(9):975. doi: 10.3390/insects16090975 (PMC12470717; doi:10.3390/insects16090975)
Supplement: Supplementary file 1 [file insects-16-00975-s001.zip › insects-3817173-supplementary.pdf]

Supplementary files

Table S1. Summary of sample sources.

Year	Location	ID	n	Plant species	Type
2008	Liaocheng	LC08	10	eggplant	open field
	Zaozhuang	ZZ08	10	cotton	open field
	Shouguang	SG08	10	tomato	greenhouse
	Dezhou	DZ08	10	cucumber	open field
	Jinan	JN08	10	cucumber	open field
2013	Liaocheng	LC13	10	eggplant	open field
	Zaozhuang	ZZ13	10	radish	open field
	Shouguang	SG13	8	tomato	greenhouse
	Dezhou	DZ13	10	eggplant	open field
	Jinan	JN13	10	eggplant	open field
2015	Liaocheng	LC15	10	eggplant	open field
	Zaozhuang	ZZ15	10	eggplant	open field
	Shouguang	SG15	10	cucumber	greenhouse
	Dezhou	DZ15	10	eggplant	open field
	Jinan	JN15	10	cotton	open field
2017	Liaocheng	LC17	10	eggplant	open field
	Zaozhuang	ZZ17	10	eggplant	open field
	Shouguang	SG17	10	muskmelon	greenhouse
	Dezhou	DZ17	10	eggplant	open field
	Jinan	JN17	10	eggplant	open field

Table S2. Pairwise F_{ST} values for all pairs of populations, all the values were insignificant ($P > 0.05$)

2008	LC	-								
	ZZ	0.0086								
	SG	0.0076	0.0039							
	DZ	0.0054	0.0063	0.0036						
	JN	0.0082	0.0089	0.0108	0.0098					
2013	LC	0.0044	0.0020	0.0048	0.0032	0.0069				
	ZZ	0.0067	0.0031	0.0029	0.0025	0.0079	-0.0003			
	SG	0.0056	0.0046	0.0032	0.0012	0.0071	0.0012	0.0002		
	DZ	0.0	0.0	0.0	0.0	0.0	-0.	0.0	0.0	

		033	092	085	050	058	0003	061	035								
	JN	0.0006	0.0028	0.0022	0.0015	0.0064	0.0005	0.0026	0.0000	-0.0008							
2015	LC	0.0116	0.0062	0.0061	0.0042	0.0124	0.0030	0.0015	0.0042	0.0075	0.0011						
	ZZ	0.0096	0.0081	0.0037	0.0023	0.0109	0.0040	0.0042	0.0034	0.0059	-0.0004	0.0006					
	SG	0.0038	0.0061	0.0066	0.0070	0.0113	0.0020	0.0053	0.0047	0.0023	0.0022	0.0082	0.0067				
	DZ	0.0027	0.0025	0.0007	0.0047	0.0086	0.0012	0.0027	0.0001	0.0034	-0.0045	0.0036	0.0035	0.0047			
	JN	0.0039	0.0026	0.0012	0.0049	0.0082	0.0009	0.0009	0.0000	0.0046	0.0003	0.0033	0.0052	0.0061	0.0017		
2017	LC	0.0041	0.0002	0.0034	0.0004	0.0091	-0.0017	0.0003	-0.0008	0.0011	-0.0029	0.0002	0.0001	0.0031	-0.0017	-0.0019	
	Z	0.	0.	0.	0.	0.	0.	0.	0.	0.	-	0.	0.	0.	0.	0.	0.

Z	0000	0065	0103	0051	0098	0033	0029	0083	0030	0.0008	0097	0098	0009	0020	0060	0034				
SG	0.0057	0.0040	0.0016	0.0069	0.0111	0.0020	0.0052	0.0059	0.0063	0.0017	0.0043	0.0098	0.0045	0.0059	0.0051	0.0049	0.0039			
DZ	0.0035	0.0009	0.0007	0.0020	0.0076	-0.0002	0.0040	-0.0007	0.0059	-0.0004	0.0002	0.0008	0.0051	0.0014	0.0001	-0.0018	0.0026	0.0008		
JN	0.0074	0.0067	0.0073	0.0051	0.0090	0.0045	0.0051	0.0064	0.0072	0.0038	0.0080	0.0088	0.0060	0.0038	0.0019	0.0023	0.0075	0.0116	0.0060	
	LC2008	ZZ	SG	DZ	JN	LC2013	ZZ	SG	DZ	JN	LC2015	ZZ	SG	DZ	JN	LC2017	ZZ	SG	DZ	JN

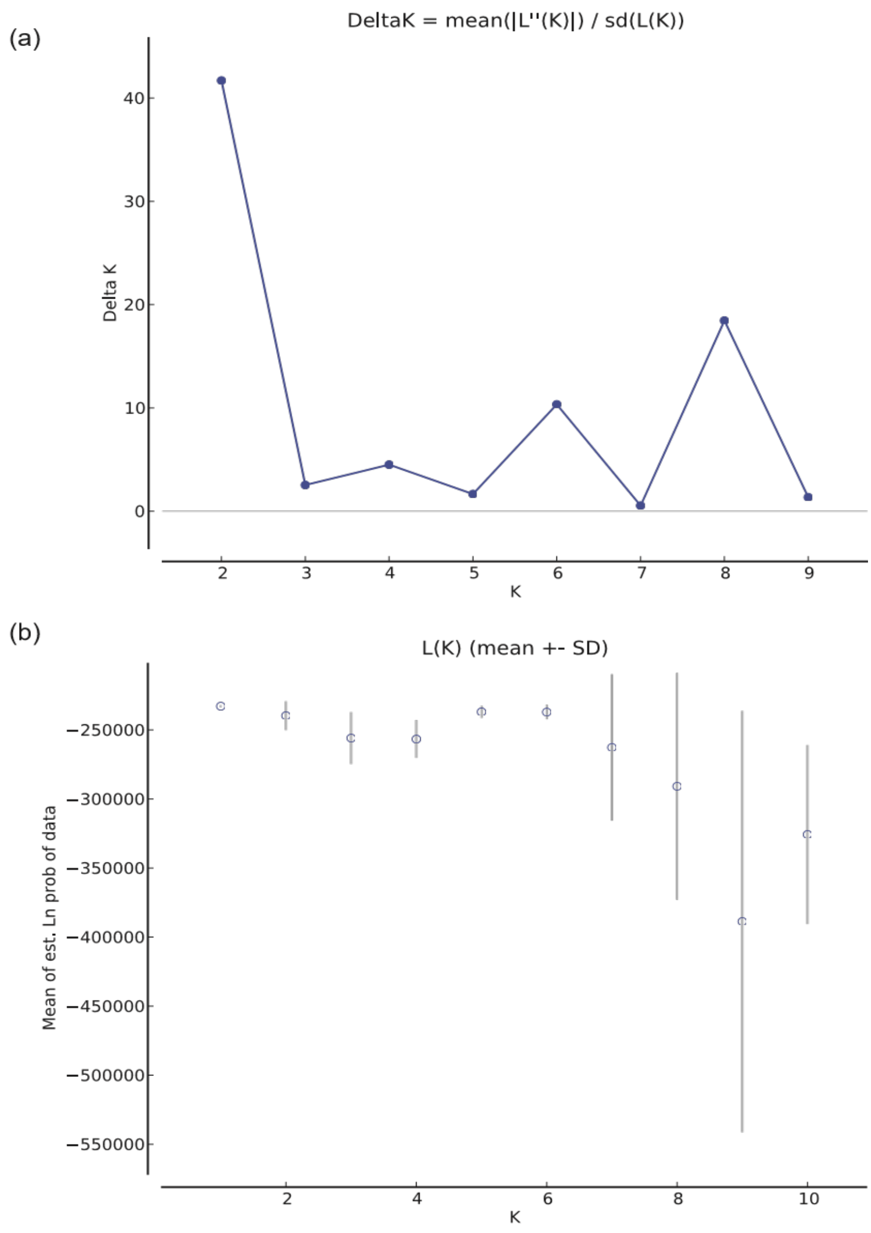

Figure S1. (**a**) Delta k of Evanno *et al.* (2005) across 10 replicates of STRUCTURE, where k = 2 is shown as the best fit of the data for the highest level of hierarchical genetic structure. (**b**) The mean lnP(D|K) and SD of 10 replicates of STRUCTURE runs for each k where the model of k = 2 is indicated as the best fit.
